# Coupling Gold Nanospheres into Nanochain Constructs for High-Contrast, Longitudinal Photoacoustic Imaging

**DOI:** 10.1021/acs.nanolett.4c00992

**Published:** 2024-05-15

**Authors:** Myeongsoo Kim, Kelsey P. Kubelick, Don Vanderlaan, David Qin, Jeungyoon Lee, Anamik Jhunjhunwala, Melissa Cadena, Robert J. Nikolai, Jinhwan Kim, Stanislav Y. Emelianov

**Affiliations:** † Wallace H. Coulter Department of Biomedical Engineering, Georgia Institute of Technology and Emory University School of Medicine, Atlanta, Georgia 30332, United States; ‡ Petit Institute for Bioengineering and Biosciences, Georgia Institute of Technology, Atlanta, Georgia 30332, United States; § School of Electrical and Computer Engineering, Georgia Institute of Technology, Atlanta, Georgia 30332, United States; ∥ Department of Biomedical Engineering, University of California Davis, Davis, California 95616, United States; ⊥ Department of Surgery, School of Medicine, University of California Davis, Sacramento, California 95817, United States

**Keywords:** photoacoustic imaging, gold nanoparticles, nanoparticle coupling, pulsed heat generation, photostability

## Abstract

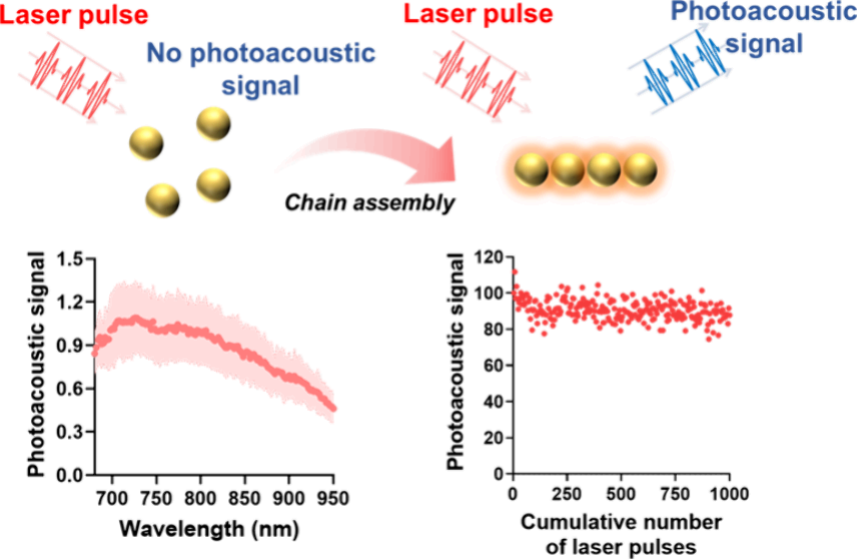

Structural parameters play a crucial role in determining the electromagnetic and thermal responses of gold nanoconstructs (GNCs) at near-infrared (NIR) wavelengths. Therefore, developing GNCs for reliable, high-contrast photoacoustic imaging has been focused on adjusting structural parameters to achieve robust NIR light absorption with photostability. In this study, we introduce an efficient photoacoustic imaging contrast agent: gold sphere chains (GSCs) consisting of plasmonically coupled gold nanospheres. The chain geometry results in enhanced photoacoustic signal generation originating from outstanding photothermal characteristics compared to traditional gold contrast agents, such as gold nanorods. Furthermore, the GSCs produce consistent photoacoustic signals at laser fluences within the limits set by the American National Standards Institute. The exceptional photoacoustic response of GSCs allows for high-contrast photoacoustic imaging over multiple imaging sessions. Finally, we demonstrate the utility of our GSCs for molecular photoacoustic cancer imaging, both in vitro and in vivo, through the integration of a tumor-targeting moiety.

Photoacoustic (PA) imaging combines the advantages of ultrasound (US) and optical imaging and has demonstrated huge benefits for noninvasive, nonionizing, and real-time imaging.^1−4^ In PA imaging, especially for in vivo applications, contrast agents must exhibit robust optical absorption within the near-infrared (NIR) window, ranging from 650 to 1300 nm, to allow deeper light penetration due to reduced optical scattering in tissue.^5,6^ There are a few endogenous absorbers in this optical window, such as (de)oxygenated hemoglobin, being widely used in monitoring spatial and functional aspects of vasculature, including hemodynamics and oxygen saturation.^7−9^ On top of that, exogenous contrast agents further enhance PA imaging contrast, facilitating the visualization of specific regions of interest through molecular targeting.^10,11^ Thus far, gold nanoconstructs (GNCs) have attracted significant attention as contrast agents for PA imaging due to their large absorption cross-section, high heat conductivity, versatile surface functionality, and cytocompatibility.^12−14^ Specifically, GNCs with high aspect ratios, such as gold nanorods (GNRs), exhibit strong PA signal and contrast due to the robust optical absorption at NIR wavelengths.^5,15,16^ However, the photodamage threshold of GNCs is inversely proportional to their aspect ratio.^17−19^ Consequently, anisotropic GNCs are susceptible to transforming into spherical shapes due to laser heating-induced atomic diffusion.^15,17,19^ This inherent lack of photothermal stability severely limits their potential in PA imaging applications for longitudinal tracking of the regions of interest throughout multiple imaging sessions, which is more likely to happen in practical and clinical scenarios.

Unlike anisotropic GNCs, gold nanospheres (GNSs) maintain their optical and structural characteristics even at laser fluences higher than those typically used in most in vivo imaging applications,^20−22^ even exceeding the limits established by the American National Standard Institute (ANSI).^23^ However, for in vivo imaging applications, the PA signal amplitude and contrast from GNSs are difficult to distinguish from endogenous molecules, especially deoxygenated hemoglobin, due to their overlap in spectrum and low absorption cross-section at NIR wavelengths.^7,21^ To enhance the NIR absorption cross-section of GNS, some previous studies employed the plasmon coupling by reducing the interparticle spacing between adjacent GNSs, thus resulting in a redshift of the peak absorption wavelength and an increase in the absorption cross-section.^24−26^ Considering remarkable photostability of GNSs, assembling them into anisotropic shape could potentially address the low photostability issue of conventional anisotropic GNCs, while exhibiting a reliable PA signal at NIR wavelengths.^24,27^ Despite the promising prospect of anisotropic structures composed of GNSs for PA imaging applications, only a few studies have been conducted due to three main reasons: (1) limited theoretical understanding of how integrating GNSs into anisotropic assemblies affects optical and PA responses, (2) challenge of achieving uniform coupling of GNSs to form an anisotropic structure, and (3) high complexity and low reproducibility in the formation of such anisotropic structures.

In this study, we conduct both theoretical and experimental investigations into the optical and thermal responses of an anisotropic assembly of GNSs into a chain-like structure, referred to as a GNS chain (GSC). Using finite-difference time-domain (FDTD) simulations, we confirmed that the GSC exhibits a higher absorption cross-section and heat transfer than a GNR and GNR chain (GRC) with similar construct dimensions. Guided by our theoretical findings, we introduced a ligand-mediated, one-step facile assembly of GNSs to create GSCs by incorporating dopamine molecules. Here, dopamine molecules not only facilitate the chain structure formation but also serve as surface coating materials through the polymerization (polydopamine), further enhancing the structural and photothermal stability of GSCs. Our results indicate that GSCs encapsulated within the polydopamine layer display robust optical absorption at NIR wavelengths through anisotropic interparticle plasmon coupling. This enhanced optical response results in a stronger PA signal amplitude and contrast compared with GNRs encapsulated within the identical polydopamine layer and pure GNRs. Moreover, our GSCs exhibit a remarkable photodamage threshold, maintaining stable PA signal generation, even at a laser fluence corresponding to the ANSI limit. Finally, by incorporating a tumor-targeting moiety, we successfully demonstrated our GSCs for both in vitro and in vivo molecular PA cancer imaging application.

In PA imaging, under pulsed laser illumination, the optical absorption of the GNCs directly contributes to PA signal generation through light-to-heat conversion and subsequent heat transfer into the surrounding medium.^5,12,28^ Therefore, when designing GNCs as PA imaging contrast agents, it is crucial to fabricate GNCs with a large absorption cross-section at NIR wavelengths to achieve high-contrast PA imaging. Considering that the size and morphology of GNCs determine their optical properties, we hypothesized that tuning the construct geometry of anisotropic GNCs could modulate optical and PA responses at NIR wavelengths. Given that three or four aspect ratios of GNCs are normally utilized for PA imaging due to the strong optical absorption within the NIR window,^11,15,29−31^ we calculated optical cross-sections, including extinction, absorption, and scattering, for three different geometry types of anisotropic GNCs with identical construct dimensions (40 nm by 120 nm), including GNR, GNR chain, and GNS chain, all embedded in water via FDTD simulations.

The computational results demonstrated that all construct geometry types exhibited a strong optical extinction peak within the NIR window, showing their suitability for high-contrast PA imaging (Figure 1a–c). At the peak extinction wavelength for each geometry, GSC exhibits 60% and 30% lower scattering percentages compared to GNR and GRC, respectively (Figure 1d and Supplementary Note 1). This indicates that when their optical density is maintained identical at their peak extinction wavelength, GSC could exhibit more efficient light-to-heat conversion compared to GNR and GRC with the lower scattering event. Furthermore, the optical absorption cross-section per single construct volume of GSC is 20% and 10% higher than that of GNR and GRC, respectively, indicating that GSC will exhibit the highest pulsed heat generation under pulsed laser illumination^32,33^ (Figure 1e and Supplementary Note 1). Moreover, GSC has 30% and 20% larger surface-to-volume ratio than GNR and GRC (Figure 1f), which promotes heat transfer into the surrounding medium compared to other constructs.^5,34,35^ Together, the results demonstrate that GSC geometry is optimally suited when designing an anisotropic GNC for achieving high-contrast PA imaging.

**Figure 1:**
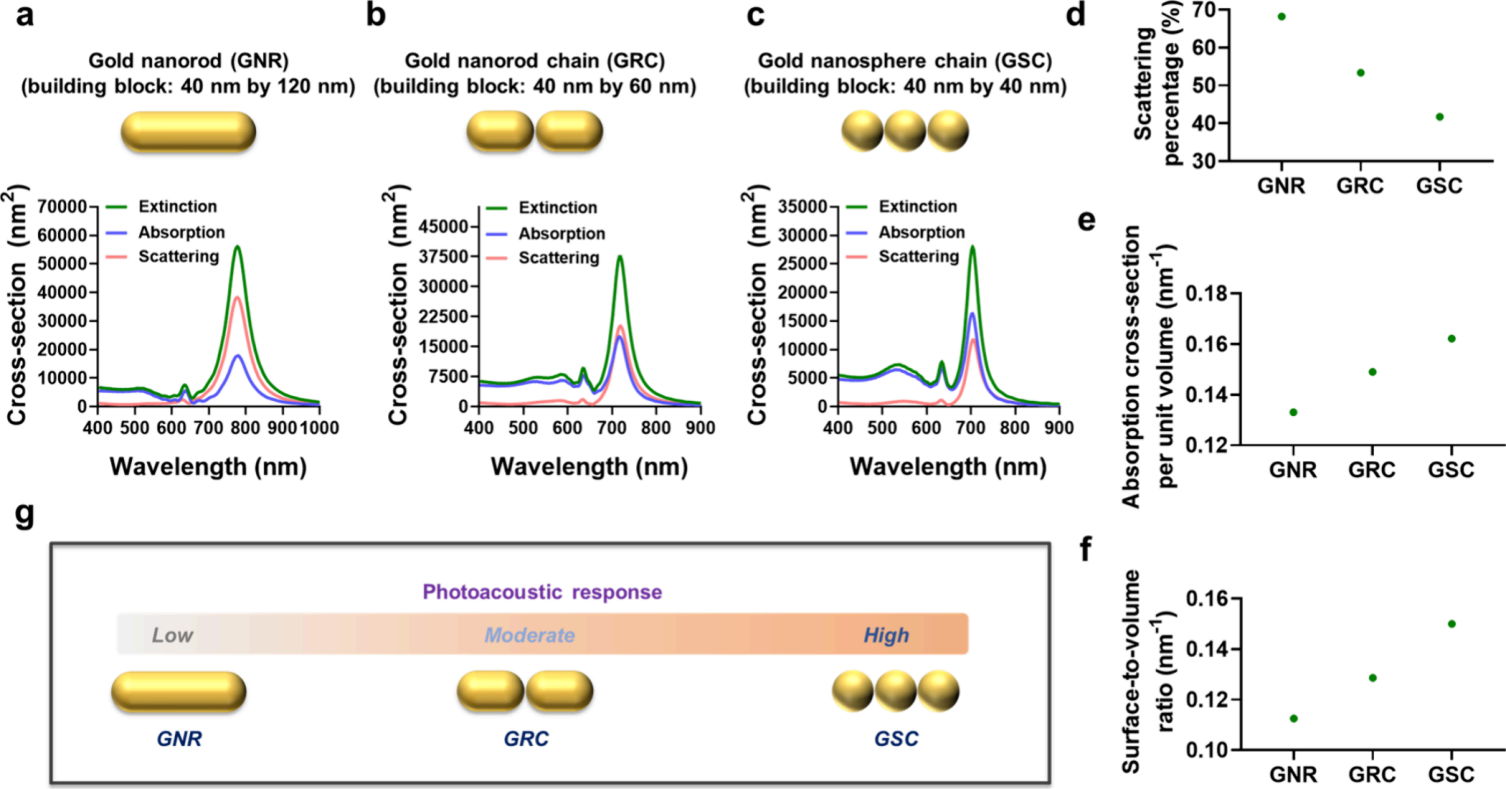
Tuning construct geometry in an anisotropic GNCs to modulate optical and PA responses at NIR wavelengths. (a–c) Calculated extinction, absorption, and scattering cross-sections of GNR, GRC, and GSC. (d, e) Estimating the scattering percentage and absorption cross-section of GNCs with different construct geometries per unit volume at the peak absorption wavelength. (f) Calculating the surface-to-volume ratio of GNCs with different construct geometries. (g) Schematic depicting the effect of the construct geometry of anisotropic GNCs on PA signal generation.

Guided by our simulation results, which indicate the promising optical and thermal properties of GSCs for high-contrast PA imaging (Figure 1), we proceeded to fabricate GSCs by incorporating GNSs into a linear chain configuration. Specifically, citrate-functionalized GNSs (40 nm)^36^ were synthesized to serve as the building block for GSC assembly (Figure 2a and b). The integration of GNSs into GSCs was initiated by electrostatic interactions between dopamine ligands and GNSs^37−39^ (Figure 2c). The addition of dopamine molecules to the GNS suspension resulted in the screening of the electrostatic repulsion between neighboring GNSs based on the electrostatic interaction between negatively charged citrate groups on GNSs and positively charged amine groups on the dopamine ligands^38,40^ (Figure S1, Supporting Information). This approach further entails the growth of a polydopamine shell layer on the surface of GSCs, enhancing their structural stability and preventing GSC disassembly.^15^

**Figure 2:**
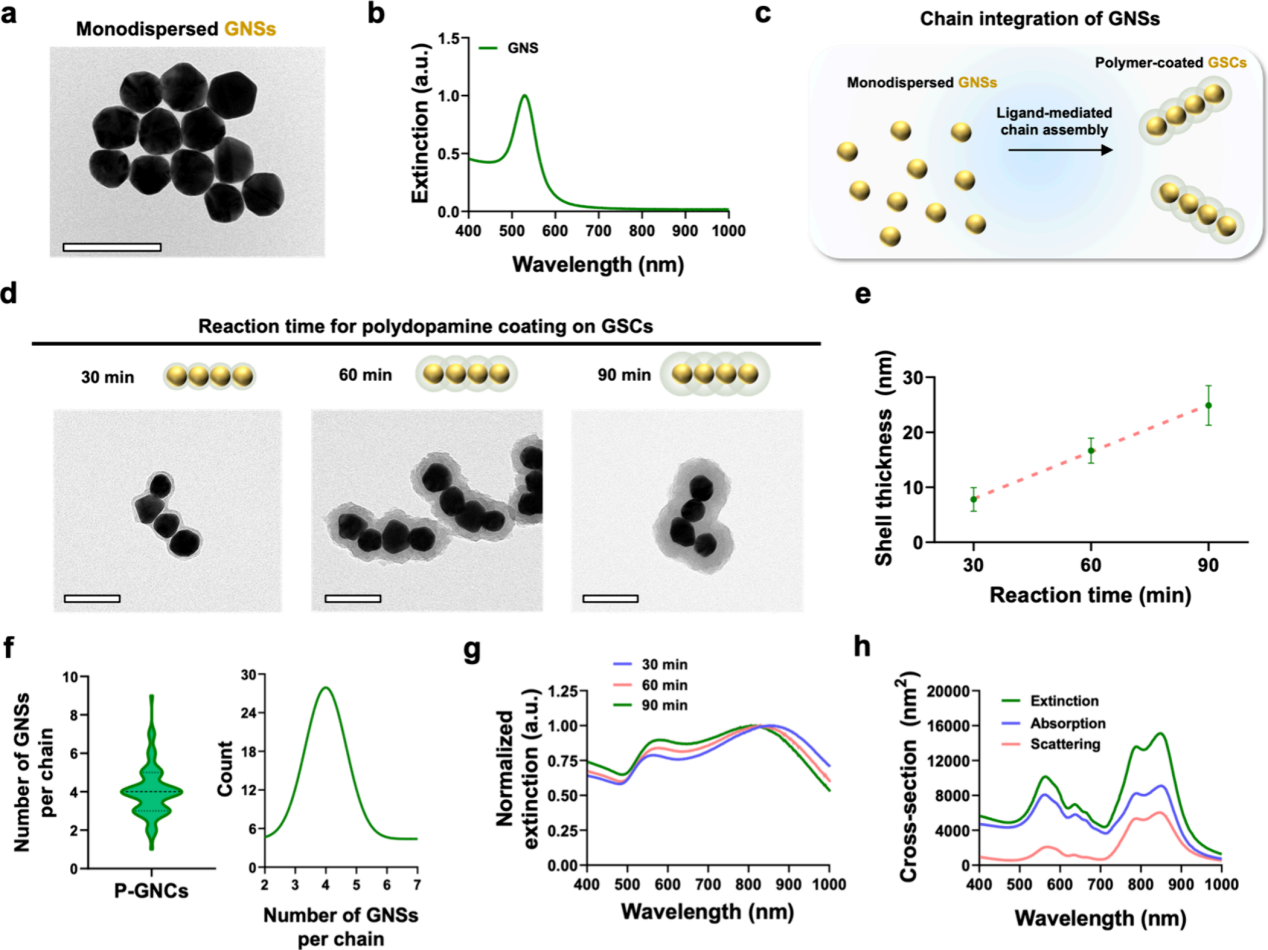
Creation of GSCs encapsulated by a polydopamine layer via the ligand-aided self-assembly of GNSs. (a, b) A TEM image and UV–vis–NIR spectrum of 40 nm-sized GNSs. (c) A schematic depicting the chain assembly of GNSs to create GSCs. The scale bar is 100 nm. (d, e) TEM images of P-GSCs versus the reaction time for dopamine polymerization and corresponding quantification of polydopamine shell thickness on GSCs (*n* = 30). The scale bars are 100 nm. (f) Estimated number of GNSs per P-GSC (*n* = 75). (g) UV–vis–NIR spectra of P-GSCs fabricated at a different reaction times. (h) Calculated extinction, absorption, and scattering cross-sections for P-GSCs. The scale bars are 50 nm. Data are presented as the mean ± standard deviation.

Given that the interaction events of dopamine ligands with GNSs at the interface of GNSs determine the chain assembly of GNSs, we adjusted the dopamine concentration in the chain assembly process from 0.5 mM to 8 mM, while keeping the GNS concentration constant at 0.35 nM. Results showed that the chain assembly of GNSs occurred beyond a dopamine concentration of 2 mM in the reaction, leading to strong optical absorption at NIR wavelengths (Figure S2). Specifically, as the dopamine concentration increased from 2 mM to 8 mM in the chain assembly, the intensity of optical absorption at NIR wavelengths slightly increased, attributed to the light absorption by the thicker polydopamine layer to light absorption.^15,41^ Furthermore, an excess amount of dopamine ligands (8 mM) led to the self-polymerization of dopamine outside the chain construct (Figure S2, Supporting Information). Therefore, we selected a dopamine concentration of 4 mM in the chain assembly process, where the GSCs exhibited the strongest optical absorption and structural homogeneity.

We then examined the impact of dopamine polymerization time on the chain assembly process while maintaining a constant dopamine concentration of 4 mM. Transmission electron microscopy (TEM) analysis showed the chain assembly process of GNSs and subsequent growth of a polydopamine shell on GSCs (Figure 2d and e). Furthermore, it was observed that GSCs consisted of an average of four GNSs with quasi-connectivity in various configurations (Figures 2f, S3, and S4, Supporting Information). UV–vis–NIR spectroscopy demonstrated that the formation of polydopamine-coated GSCs (P-GSCs) led to an increased optical extinction at NIR wavelengths (Figure 2g). Moreover, the reproducibility of P-GSC synthesis was confirmed by observing consistent optical properties across three different batches of P-GSCs (Figure S5, Supporting Information). Next, to calculate the scattering percentage of P-GSCs at NIR wavelengths, we conducted an FDTD analysis within the 400–1000 nm spectral range (Figure 2h). The simulation result showed that P-GSCs exhibited strong optical responses within this spectral range, displaying an optical cross-section in absorption approximately 50% higher than that in scattering at the peak absorption wavelength (Figure 2h). It should be noted that a minor disparity was observed in peak positions and broadening between the experimental and computational spectra. This variance arises from the experimental data obtained via UV–vis–NIR spectroscopy, which encompasses more variations in GNS number/size, interparticle distances, chain–chain optical interactions, and a broader range of chain configurations within the ensemble (Figure S6, Supporting Information). Taken together, these findings underscore the potential of our P-GSCs as exogenous contrast agents for achieving high-contrast PA imaging.

Based on the strong optical absorption of P-GSCs in the NIR window (Figure 2g), we initially characterized PA signal generation at 800 nm from P-GSCs with different polydopamine layer thicknesses to investigate the influence of the polydopamine layer in P-GSCs on the PA signal amplitude. As the thickness of the polydopamine layer on GSCs increased, the PA signal amplitude from P-GSC slightly increased, suggesting that a thicker polydopamine layer in P-GSCs contributes to enhanced optical absorption (Figures 3a and S7, Supporting Information). However, although a thicker polydopamine layer on chain constructs led to stronger PA signal generation, the PA signals did not exhibit statistically significant differences (Figure 3a), suggesting that the primary determinant of PA responses from P-GSCs is the GSC core, rather than the polydopamine shell layer. Furthermore, P-GSCs exhibited strong PA signal amplitudes within the NIR spectral region, particularly at 800 nm, which is aligned with their optical absorption spectrum (Figures 2g and S8, Supporting Information). Additionally, P-GSCs were detectable even at a concentration of 6.25 pM, exhibiting a linear increase in PA signal and contrast with higher particle concentrations (Figure 3b).

**Figure 3:**
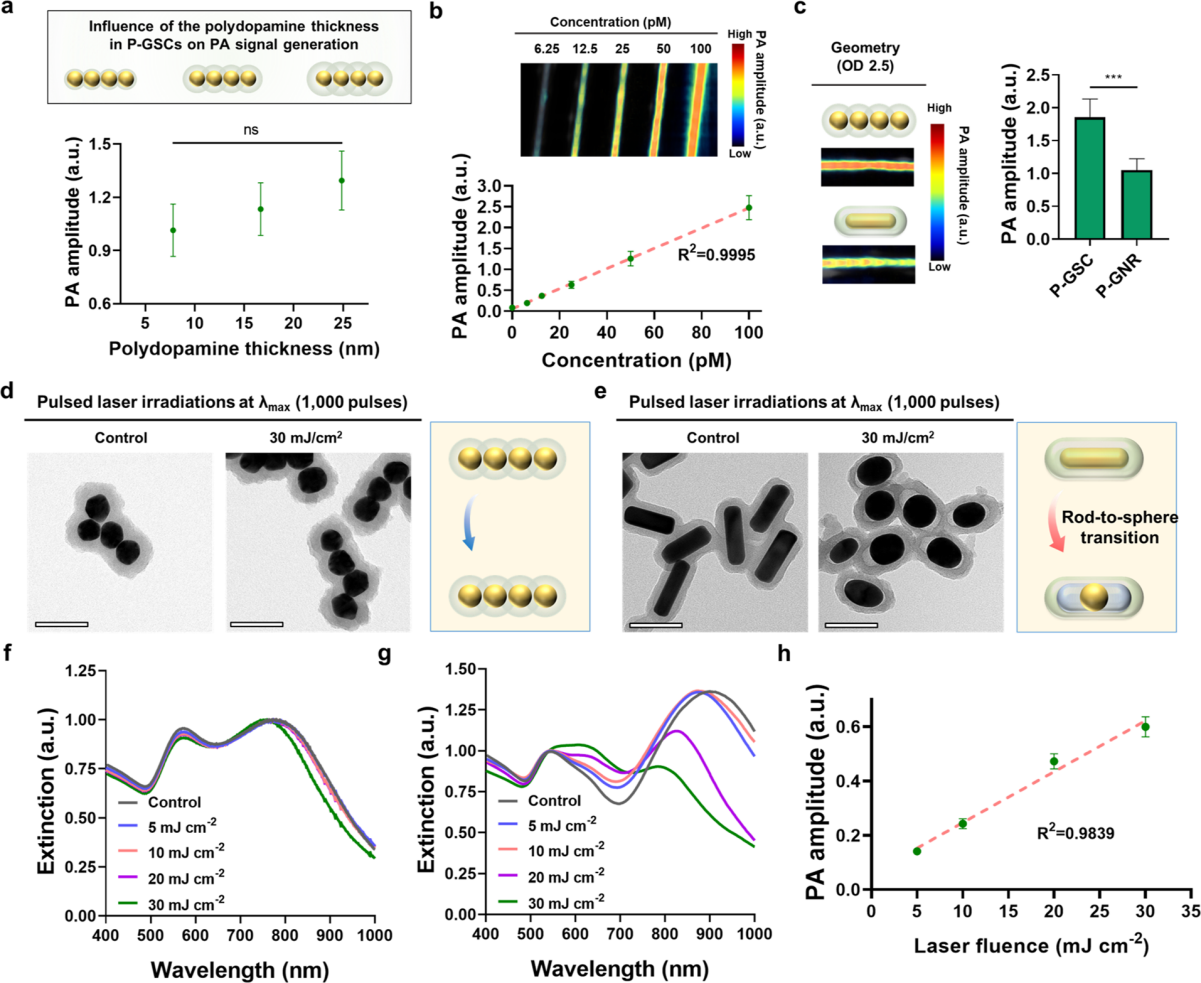
Analysis of PA signal generation from P-GSCs. (a) PA signal amplitude of P-GSCs with various thicknesses of the polydopamine layer (50 pM, *n* = 5). (b) PA signal generation from P-GSCs at different construct concentrations ranging from 0 to 100 pM (*n* = 5). (c) PA signal generation from P-GSCs and P-GNRs at their peak absorption wavelength (OD 2.5, *n* = 5). (d, e) TEM images of P-GSCs and P-GNRs after pulsed laser illumination for 1000 pulses at a laser fluence of 30 mJ cm^–2^. Scale bars are 100 nm. (f, g) UV–vis–NIR spectra of P-GSCs and P-GNRs after pulsed laser illumination for 1000 pulses at different laser fluences. (h) PA signal generation from P-GSCs at different laser fluences (*n* = 250). Data are presented as the mean ± standard deviation. The statistical analyses for (a) and (c) were conducted using one-way analysis of variance (ANOVA) using Tukey’s posthoc test and a two-tailed Student’s *t* test, respectively. The statistically nonsignificant difference is represented as ns. The statistically significant difference is represented as the asterisk (***: *p* < 0.001). The imaging experiments were repeated independently three times, and similar imaging results were obtained.

Next, PA signal generation from P-GSCs was compared with that from polydopamine-coated GNRs (P-GNRs), both having a similar construct dimension and polydopamine layer thickness (Figure S9, Supporting Information). We measured their PA signal amplitude and contrast at their peak absorption wavelength (800 and 900 nm, respectively) while maintaining the solvent (water), volume of each GNC solution, laser fluence (10 mJ cm^–2^), and optical density (OD). Results showed that P-GSCs exhibited an approximately 80% higher PA amplitude compared to P-GNRs (Figure 3c). FDTD simulation analysis revealed that P-GSCs had an approximately 37% lower scattering percentage at their corresponding peak absorption wavelength compared to P-GNRs (Figure S10, Supporting Information). This lower scattering percentage indicates that P-GSCs can more effectively absorb incident laser light, thus producing a stronger PA signal amplitude and contrast compared to P-GNRs. In addition to the lower scattering percentage of P-GSCs, the larger surface-to-volume ratio of P-GSCs could allow pulsed heat transfer more effectively into the surrounding medium, resulting in a higher PA signal amplitude.^5,34,35^ Moreover, P-GSCs produced an approximately 550% higher PA amplitude compared to pure GNRs without a polydopamine layer, which is representative of conventional plasmonic PA contrast agents (Figure S11, Supporting Information). Collectively, results demonstrate that our P-GSCs possess the potential to outperform conventional plasmonic contrast agents for high-contrast PA imaging applications.

The photodamage threshold of GNCs is a critical factor in performing PA imaging across multiple imaging sessions. Therefore, we characterized a photodamage threshold of P-GSCs in comparison to that of P-GNRs under pulsed laser illumination at varying laser fluences. While P-GNRs underwent a shape transition to spherical shapes and exhibited a decreased intensity in NIR light absorption at a laser fluence of 20 mJ cm^–2^, P-GSCs maintained their structural and optical properties even at a laser fluence of 30 mJ cm^–2^ (Figures 3d–g and S12, Supporting Information). Due to the exceptional photostability of P-GSCs, they displayed a linear increase in PA signal amplitude with increasing laser fluence (Figure 3h and Figure S12, Supporting Information). Notably, according to the ANSI, the maximum allowable laser fluence for clinical applications is approximately 30 mJ cm^–2^ at 800 nm wavelength.^23^ Considering that P-GSCs can produce stable PA signal generation over 1000 pulses at 30 mJ cm^–2^, the exceptional photostability of P-GSCs highlights their potential to outperform traditional plasmonic PA contrast agents, including gold nanorods,^5,42,43^ gold nanostars,^44^ gold nanoplates,^45^ and silica-coated GNRs,^31,46^ for PA imaging applications required for longitudinal stability of imaging contrast over multiple imaging sessions (Figure S13 and Table S1, Supporting Information).

Next, we evaluated the imageability of our P-GSCs for biomedical ultrasound-guided PA (US/PA) imaging applications, specifically cancer imaging, both in vitro and in vivo. We first functionalized P-GSCs with cyclic Arg-Gly-Asp (cRGD) tripeptides to specifically recognize integrin α_v_β_3_ receptors that are normally overexpressed in a variety of cancer cells, including MDA-MB 231^47,48^ (Figure 4a). We chose the cyclic RGD peptide for the functionalization of P-GSCs because the head-to-tail cyclization of peptides results in better potency, specificity, and chemical stability compared to linear RGD peptides.^47^ The changes in surface charges of P-GSCs for each step of the cRGD coupling process confirmed the successful functionalization of cRGD ligands onto the P-GSCs (Figure S14, Supporting Information).

**Figure 4:**
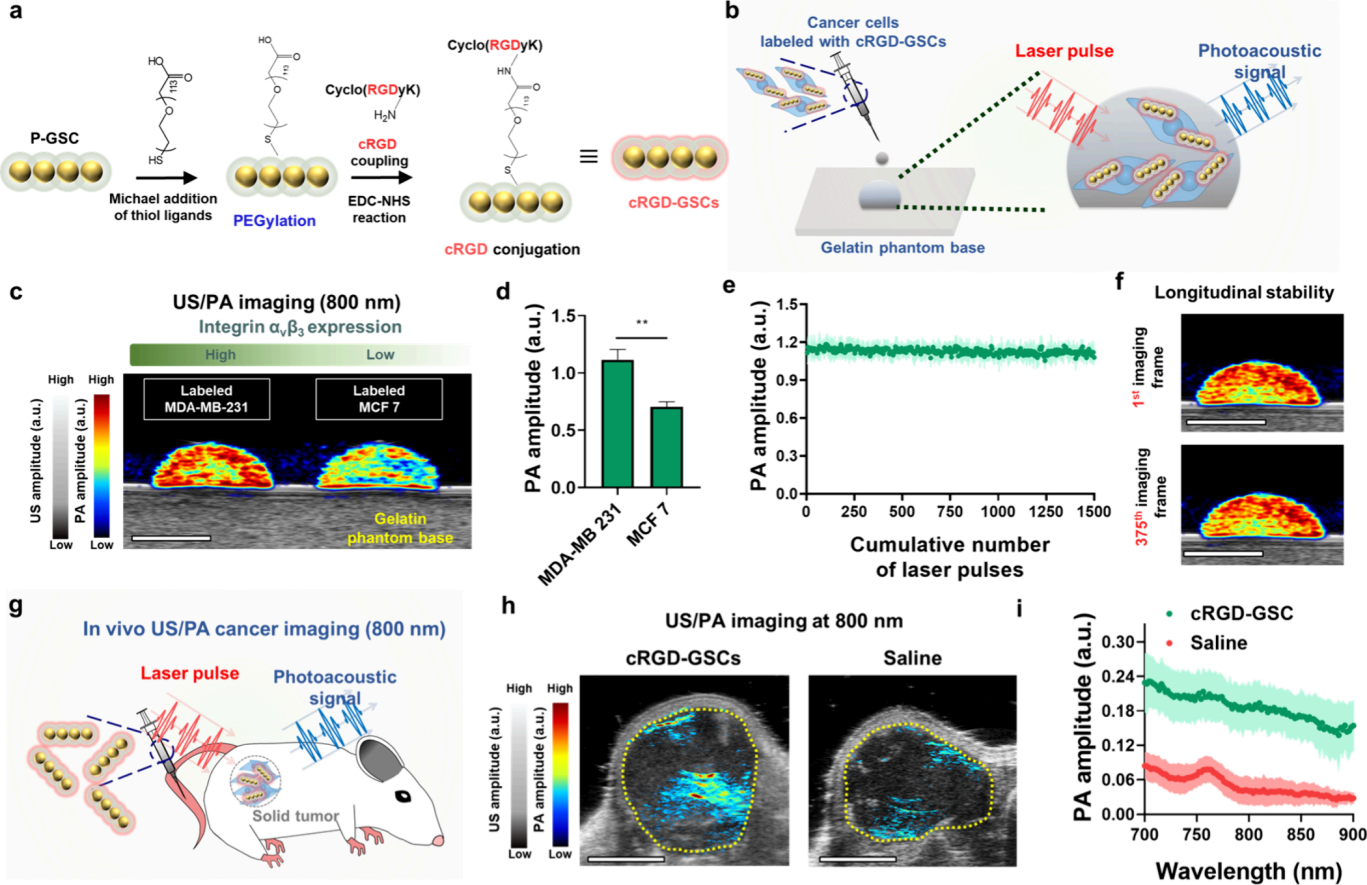
In vitro and in vivo US/PA cancer imaging utilizing cyclic RGD-coupled P-GSCs. (a) Schematic depicting a strategy to functionalize cyclic RGD peptides onto P-GSCs. (b) Schematic of in vitro US/PA cancer cell imaging through the labeling of MDA-MB 231 or MCF 7 cancer cells with cRGD-coupled P-GSCs (cRGD-GSCs). (c) US/PA images of the labeled cells at 800 nm. (d) Corresponding PA signal amplitudes at 800 nm. (e) PA signal generation from the labeled MDA-MB 231 cells for 1500 laser pulses at 800 nm. For in vitro imaging quantification results, data are presented as the mean ± standard deviation. (f) US/PA image of labeled MDA-MB 231 cells at the first and 375th imaging frame. The images were obtained at 800 nm. (g) Schematic of *in vivo* PA cancer imaging through the tail-vein injection of cRGD-GSCs. (h) US/PA images of the tumor site with or without the systemic delivery of cRGD-GSCs. (i) Corresponding quantifications of PA signals from the tumor sites (*n* = 3). Data are presented as the mean ± standard deviation. The scale bars are 4 mm. The statistical analysis for (d) was conducted using a two-tailed Student’s *t* test. The statistically significant difference is represented as the asterisk (**: *p* < 0.01). The imaging experiments were repeated independently three times, and similar imaging results were obtained.

For a demonstration of the feasibility of in vitro cancer imaging with target specificity, we set MDA-MB 231 and MCF 7 cancer cells as the positive and negative control group, respectively. This choice was made because MDA-MB 231 cells exhibit a higher level of expression of integrin α_v_β_3_ compared to MCF 7.^49,50^ For in vitro US/PA imaging, we labeled both cancer cells using cRGD-coupled P-GSCs (cRGD-GSCs) at a construct concentration of 30 pM. This construct concentration in the cell labeling process did not show any significant toxicity to the cells (Figure S15, Supporting Information). The labeled MDA-MB 231 cells exhibited an approximately 60% higher PA signal and contrast at an 800 nm wavelength compared to the labeled MCF 7 cells (Figure 4b–d). The labeled cancer cells exhibited excellent PA imaging stability over 1500 pulses at a laser fluence of 10 mJ cm^–2^, which corresponds to 375 imaging frames (Figure 4e,f). Moreover, labeling MDA-MB 231 cancer cells with cRGD-GSCs resulted in an approximately 60% higher PA signal amplitude compared to that with scrambled-cRGD-conjugated P-GSCs (cyclic RAD), cRGD-GSCs with free cRGD ligands (inhibition control), and PEGylated P-GSCs (Figure S16, Supporting Information). This enhancement in the PA signal suggests that cRGD coupling to GSCs improves their target specificity to MDA-MB 231 cells by facilitating recognition of the integrin α_v_β_3_ receptors expressed on the cancer cells and promoting cellular internalization. Together, results confirm the capability of cRGD-GSCs for molecular cancer cell imaging with high imaging contrast and longitudinal stability in the NIR window.

Finally, we demonstrated our cRGD-GSCs as an imaging contrast agent for in vivo PA cancer imaging. To validate the imageability of cRGD-GSCs in vivo, we conducted a preliminary study by dispersing cRGD-GSCs in Matrigel and subcutaneously injected them into healthy mice. Subsequently, we performed US/PA imaging of the injected cRGD-GSCs at the 680–900 nm wavelengths (Figure S17a, Supporting Information). While Matrigel exhibited negligible PA amplitude and contrast, mice that locally received the local administration of cRGD-GSCs showed distinct PA amplitude and contrast with imaging stability (Figure S17b and d, Supporting Information), due to the exceptional PA response and stability as previously shown in Figure 3. Next, we proceeded to validate the potential of our cRGD-GSCs for PA cancer imaging. Mice with MDA-MB 231 tumors received systemic injections of cRGD-GSC or saline, followed by US/PA imaging 24 h postinjection (Figure 4g). The tumor site in mice receiving cRGD-GSCs exhibited an approximately 300% higher PA amplitude compared to the mice receiving the saline solution (Figure 4g–i). Results conclusively demonstrate the potential of our cRGD-GSCs as a PA imaging contrast agent for high-contrast, continuing PA imaging applications in vivo.

We have created a chain construct composed of GNSs as a PA imaging contrast, designed for high-contrast, reliable PA imaging across multiple sessions, particularly for in vivo applications. Theoretical calculations revealed that sphere-based chain constructs outperform other geometries in pulsed heat generation and heat transfer, making them ideal for efficient PA imaging. Leveraging these insights, we fabricated P-GSCs using a straightforward one-step process, achieving strong optical responses at NIR wavelengths due to interparticle plasmon coupling, allowing P-GSCs to provide high PA signal and contrast, detectable at picomolar concentrations, and outperform conventional plasmonic agents like GNRs. Importantly, P-GSCs sustain PA signal generation within the ANSI laser fluence limits, validating their superiority for longitudinal, high-contrast PA imaging. We tracked the systemic delivery and tumor accumulation of functionalized P-GSCs, showcasing their potential for advanced PA imaging applications.

Our P-GSCs present multiple advantages over traditional plasmonic contrast agents for PA imaging. First, their chain morphology affords a higher absorption efficiency, potentially enhancing incident laser light absorption. Second, P-GSCs boast greater photostability, maintaining consistent PA signals even at laser fluences near the ANSI limit. Third, their large surface-to-volume ratio promotes effective heat transfer, resulting in stronger PA signals. Additionally, P-GSCs absorb strongly at 800 nm, a wavelength where common endogenous absorbers like hemoglobin show minimal interference, enabling clearer in vivo imaging and differentiation of target areas from background signals. Lastly, P-GSCs benefit from a straightforward, one-step synthesis process, offering superior consistency and reduction of batch variations. This simplifies scaling and translational research compared to the complex and variable preparation of traditional anisotropic GNCs, marking a significant advancement for PA imaging applications. However, a potential limitation of our P-GSC system is its suitability for multiplexing in bioimaging, attributed to broad optical absorption. Future work could refine structural parameters, such as GNS size, chain length, NS composition, and polymer layer characteristics, to tailor chain-shaped PA contrast agents for specific biomedical imaging needs.

## Data Availability

The data that support the plots within this paper and other findings of this study are available within the manuscript and the Supporting Information. Additional data related to the study can be requested from the corresponding author.

## Supporting Information Available

Experimental details and supplementary figures, including construct synthesis, characterization of P-GSCs, P-GNRs, and GNRs, experimental setup, computational analysis of optical cross-sections of GNCs with different construct geometries, cell viability test, quantification of PA signals from cancer cells labeled with P-GSCs with different surface ligand moieties, and in vivo PA signal generation from P-GSCs; Figures S1–17 and Table S1 (PDF)
