# Supplementary material for: Coupling Gold Nanospheres into Nanochain Constructs for High-Contrast, Longitudinal Photoacoustic Imaging
Source: Nano Lett. 2024 May 15;24(24):7202–10. doi: 10.1021/acs.nanolett.4c00992 (PMC11194844; doi:10.1021/acs.nanolett.4c00992)
Supplement: Supplementary file 1 — nl4c00992_si_001.pdf [file nl4c00992_si_001.pdf]

Supporting Information

Coupling gold nanospheres into nanochain constructs for high-contrast, longitudinal photoacoustic imaging

Myeongsoo Kim[1,2], Kelsey P. Kubelick[1,3], Don Vanderlaan[1,3], David Qin[1], Jeungyoon Lee[3], Anamik Jhunjhunwala[1], Melissa Cadena[1], Robert J. Nikolai[1], Jinhwan Kim[4,5,], and Stanislav Y. Emelianov[1,2,3,*]*

[1]Wallace H. Coulter Department of Biomedical Engineering, Georgia Institute of Technology and Emory University School of Medicine, Atlanta, GA 30332, USA

[2]Petit Institute for Bioengineering and Biosciences, Georgia Institute of Technology, Atlanta, GA 30332, USA

[3]School of Electrical and Computer Engineering, Georgia Institute of Technology, Atlanta, GA, 30332, USA

[4]Department of Biomedical Engineering, University of California Davis, Sacramento, CA 95616, USA.

[5]Department of Surgery, School of Medicine, University of California Davis, Sacramento, CA 95817, USA.

*Corresponding Authors: Stanislav. Y. Emelianov (stas@gatech.edu) and Jinhwan Kim (jjnkim@ucdavis.edu)

Supplementary Note 1.

1. Calculation of scattering percentages of GNCs with different construct geometries while maintaining constant aspect ratio.

Under pulsed laser illumination, different GNCs can interact with incident laser photons through optical absorption and optical scattering[1–3]. The optical absorption by the GNCs can primarily contribute to photoacoustic (PA) signal generation. Optical density of GNCs measured from UV-vis-NIR spectroscopy represent the optical extinction that is associated with the extinction cross-section (σ_{ext}) of the GNCs. Therefore, to achieve efficient PA conversion by GNCs at a specific optical density in a surrounding medium, the absorption cross-section (σ_{abs}) of the GNCs should be maximized while minimizing the contribution of their scattering cross-section (σ_{sca}) to the total optical extinction cross-section[2,4]. To predict optical response of GNCs with different geometries, including gold nanorod (GNR), gold nanorod chain (GRC), and gold nanosphere chain (GSC), we carried out a finite-difference time-domain (FDTD) simulation. In this simulation, we maintained the construct dimension as 40 nm (width) by 120 nm (length) to keep a constant aspect ratio and set the surrounding environment for all GNCs as water. The values in Johnson and Christy were utilized as optical characteristics of gold for computational analysis[5]. The scattering percentage was calculated as follows[6]:

$$Scattering\ percentage\ (\%) = \frac{\sigma_{sca}}{\sigma_{ext}} \times 100$$

2. Photoacoustic signal generation from GNCs.

When exogenous contrast agents with nanoscale diameters are exposed to pulsed laser illumination, laser light is dominantly absorbed by the nanoscale contrast agents rather than the surrounding medium[1–3]. Subsequently, during the laser illumination, pulsed heat leakage from the exogenous contrast occurs. Owing to the time-dependent thermal leakage, PA signal generation from exogenous contrast agents is determined by pulsed heat generation through light-to-heat conversion and subsequent heat transfer into the surrounding environment[1–3]. Therefore, when designing exogenous contrast agents for PA imaging applications, optical and thermal characteristics of the contrast agents must be engineered to achieve high-contrast PA imaging. Since structural parameters, such as size, aspect ratio, and construct geometry, determine photothermal responses of GNCs at near-infrared (NIR) wavelengths[4,6], we tried to investigate the photothermal properties by tuning the construct geometry of anisotropic GNCs with identical aspect ratio of three, such as GNR, GRC, and GSC, while maintaining their construct dimension identical (40 nm by 120 nm).

3. Effect of heat transfer property of GNCs on PA signal generation

In Fourier`s law of thermal conduction (one-dimensional) through a GNC, the rate of heat transfer, i.e., heat flux density, is described as below[7]:

$$Q = -kA\frac{\Delta T}{\Delta x} \qquad (1)$$

Where Q is the heat flux density, k is the heat conductivity of the GNC (318 W $m^{-1}K^{-1}$), A is the surface area of the GNCs, ΔT is the temperature variation of the construct surrounded by a medium under laser illumination, and Δx is the length from the GNC, respectively. In our simulation, all different GNCs had an identical surface area as 15079 nm^2.

Under pulsed laser illumination, the temperature increase of the GNC is calculated as follows[8,9]:

$$\Delta T \propto \frac{\sigma_{abs} F}{V\rho c} \qquad (2)$$

Where σ_{abs}, F, V, ρ, and c represent the absorption cross-section of the GNC, volume of the GNC, laser fluence, the density of the GNC, and heat capacity of the GNC, respectively. When GNCs with different construct geometries are illuminated by laser pulses at the identical laser fluence, the temperature increase on each GNC is directly proportional to the absorption section per unit construct volume because the density and heat capacity of the GNCs rely on the material characteristics of gold. ρ_{gold} and c_{gold} are 19.32 g cm^{-3} and 0.126 J $g^{-1}K^{-1}$, respectively. Therefore, the heat flux density can be calculated based on the equation (1) and (2) as follows:

$$Q \propto \frac{\sigma_{abs}}{V} \qquad (3)$$

Moreover, given that the heat generation from GNCs under pulsed laser illumination can be transferred to the surrounding medium in a three-dimensional direction, the transfer of heat pulses is determined by the surface-to-volume ratio for each GNC[2,3,10,11]. Collectively, since PA signal generation from GNC solutions can rely on pulsed heat generation and heat transfer properties of each GNC, we calculated absorption cross-section per unit construct volume and surface-to-volume ratio for each type of GNCs.

Supplementary Note 2. Synthesis and characterization of P-GSCs.

Synthesis of P-GSCs. To create P-GSCs, we first created 40 nm-sized GNSs via seed-mediated growth of 13 nm-sized GNSs[12]. First, we synthesized 13 nm-sized GNSs via Turkevich method[13]. In detail, 50 mL of 1 mM gold (III) chloride trihydrate ($HAuCl_4 \cdot 3H_2O$, Sigma Aldrich) aqueous solution was heated to 100 °C under magnetic stirring. To this solution, 5 mL of 38.8 mM sodium citrate (Sigma Aldrich) aqueous solution was rapidly injected, followed by stirring for 20 minutes and cooling down to room temperature.

To synthesize 40 nm-sized GNSs, we carried out a seed-mediated growth of 13 nm-sized GNSs as previously reported[14]. Specifically, GNSs (13 nm) were re-dispersed in 80 mL of deionized water, followed by heating up to 90 °C under magnetic stirring. To this solution, we added 0.4 mL of 25 mM $HAuCl_4 \cdot 3H_2O$ aqueous solution and 0.8 mL of 60 mM sodium citrate aqueous solution, followed by stirring for 30 minutes. We repeated the addition process of the $HAuCl_4 \cdot 3H_2O$ and sodium citrate solutions ten times to get 40 nm-sized GNSs.

To create P-GSCs, 12 mL of 40 nm-sized GNSs (0.35 nM) was mixed with 24 mL of 10 mM Tris-HCl buffer solution (pH 8.5). The concentration of GNSs was determined by Beer-Lambert law. To this solution, we rapidly added 3 mL of dopamine hydrochloride (Sigma Aldrich) aqueous solution (4 mM), followed by sonication for 3, 10, 30, and 90 minutes, to form different thicknesses of polydopamine layer on the GSCs. The products were then washed with deionized water via centrifugation twice (6,000 rpm, 5 min) and then re-dispersed in deionized water for further characterization, such as TEM, UV-vis-NIR spectroscopy, and PA analysis.

Synthesis of P-GNRs. GNRs with different construct dimensions (35 nm by 100 nm & 35 nm by 135 nm) were created based on a binary surfactant-assisted seeded growth approach[15]. To create 2 nm-sized gold seeds, 0.6 mL of 0.1 M sodium borohydride (Sigma Aldrich) was rapidly added to 10 mL of 0.25 mM $HAuCl_4 \cdot 3H_2O$ and 0.1 M CTAB (VWR Life Science) aqueous solutions under magnetic stirring, followed by stirring for 1 minute. To create GNRs, 0.9 g of CTAB and 0.1234 g of sodium oleate (TCI America.) were dissolved in 50 mL of DI water, followed by adding 2.4 mL (for the dimension of 35 nm by 135 nm) or 4.8 mL (for the dimension of 35 nm by 100 nm) of 4 mM silver nitrate (Sigma Aldrich) aqueous solution. The mixture was kept undisturbed for 10 minutes and 25 mL of 1 mM $HAuCl_4 \cdot 3H_2O$ aqueous solution was added to the mixture, followed by stirring for 90 minutes. To this mixture, 0.21 mL of 0.1M hydrocholoride (Sigma Aldrich) aqueous solution was added and the solution was magnetically stirred for 30 minutes. To initiate the seed-mediated growth of 2nm-sized gold seeds, we added 0.125 mL of 64 mM L-ascorbic acid (Sigma Aldrich) aqueous solution and 0.01 mL of the seed particles sequentially to the mixture under magnetic stirring. After 1 minute of stirring, the mixture was placed at 30 ^{o}C overnight. The products were isolated by centrifugation (9,000 rpm, 15 minutes) and then dispersed in 6 mL of 0.16 mM SH-PEG-COOH (Molecular weight 5k, Laysan Bio, Inc) aqueous solution for the subsequent PEGylation. After 16 hours of PEGylation under magnetic stirring, the PEGylated GNRs were dispersed in 6 mL of 10 mM Tris-HCl buffer solution (pH. 8.5) For the polydopamine coating process, 2 mL of PEGylated GNRs was re-dispersed in 6 mL of 10 mM Tris-HCl buffer solution (pH 8.5). To this solution, we added 0.5 mL of dopamine hydrochloride aqueous solution (4 mM) and stirred the mixture for 60 minutes. After the reaction, P-GNRs were washed with deionized water via centrifugation twice (6,000 rpm, 5 min) and then re-dispersed in deionized water for further characterization.

Characterization for GNCs. Transmission electron microscopy (HT 7700, Hitachi) was utilized to characterize the morphology of GNCs. The optical characteristics of GNCs were characterized by a UV-vis-NIR spectrophotometer (Evolution 220, Theormoscientific). The surface charge change of P-GSCs in the functionalization of cyclic RGD peptides was measured using a dynamic light scattering instrument (Zetasizer Nano ZS, Malvern Instruments Ltd.)

Characterization for PA signal generation from GNCs. To characterize PA signals from GNC solutions, polyethylene tubes (BD Intramedic) were held in a 3D-printed scaffold. We added 40 μL of GNCs in each tube and placed the tube phantom in the Vevo2100/LAZR imaging system (Fujifilm VisualSonics Inc.). US/PA imaging was carried out using an LZ250 ultrasound transducer for *in vitro* experiments. The laser source was a Q-switched Nd.YAG-pumped optical parametric oscillator (OPO) laser (pulse duration = 7 nanoseconds, frame rate = 20 Mz). For PA imaging, the tube phantoms were irradiated under laser pulses at 10.5 mJ cm^{-2} within the 680-960 nm spectral range with 2 nm intervals and a persistence of 3. B-mode and PA-gain were set as 40 dB and 18 dB, respectively.

Numerical simulation for P-GSCs and P-GNRs. The 3D models for the simulation of P-GSC and P-GNR were designed based on TEM analysis for both GNCs. Since P-GSCs and P-GNRs were covered with a polydopamine layer, the refractive index of the surrounding medium was set as a value of polydopamine reported in the previous literature[16]. The optical extinction, absorption, and scattering cross-section of P-GSCs were calculated by tuning the number and arrangement of GNSs in P-GSCs at varied light polarization angles and taking an average cross-section value. A total-field/scattered-field source with a spectral pulse from 400 – 1000 nm was utilized.

Characterization for PA signals from P-GSCs, P-GNRs, and pure GNRs. To characterize

PA signal generation from different GNCs, including P-GSCs, P-GNRs, and pure GNRs, polyethylene tubes (BD Intramedic) were prepared in a 3D-printed scaffold. Different GNCs (50 μL) were added in each tube and placed the scaffold with tubes in the Vevo2100/LAZR imaging system (Fujifilm VisualSonics Inc.). US/PA imags were acquired using an LZ250 ultrasound transducer. The laser source was a Q-switched Nd:YAG-pumped optical papametric oscillator (OPO) laser system (pulse duration: 7 ns, pulse frequency: 20 Hz). For PA experiments, the tubes were irradiated under laser pulses at the corresponding wavelength of peak optical absorption for P-GSCs, P-GNRs, and pure GNRs with a laser fluence of approximately 10 mJ cm^{-2}.

Characterization for a photodamage threshold of P-GSCs and P-GNRs. To characterize the photodamage threshold of P-GSCs and P-GNRs, PA signal from each GNC was acquired at different laser fluences by using the Vevo2100/LAZR imaging system (Fujifilm VisualSonics Inc.), which integrates a nanosecond Nd:YAG laser (OPOTek). Tube phantoms containing each GNC solution were irradiated by using a collimated beam of uniform laser fluence. Orientation of the laser propagation was orthogonal to the axis of tube phantoms for the PA imaging. The laser fluence was adjusted from 5 mJ cm^{-2} to 30 mJ cm^{-2} by modulating Q-switch delay time and neutral density filters. P-GSCs and P-GNRs were irradiated by laser pulses at their corresponding maximum absorption wavelength.

Coupling of cyclic RGD or RAD tripeptides to P-GSCs. To couple tumor-targeting RGD moiety to P-GSCs, P-GSCs were functionalized with carboxyl group-terminated PEG molecules. Specifically, 1 mL of P-GSCs (850 pM) in 10 mM Tris-HCl buffer solution was mixed with 0.2 mL of 1 mM SH-PEG-COOH aqueous solutions, followed by shaking overnight. The PEGylated P-GSCs were washed with deionized water three times via centrifugation (6,000 rpm, 10 minutes). To couple cRGD or cRAD peptides to P-GSCs, 1 mL

of PEGylated P-GSCs in deionized water was mixed with 0.05 mL of 20 mM 1-(3-Dimethylaminopropyl)-3-ethylcarbodiimide (EDC, Alfa Aesar) and 0.05 mL of 20 mM N-Hydroxysulfosuccinimide Sodium Salt (Sulfo-NHS, bioWORLD), followed by shaking for 30 minutes. After shaking, EDC/NHS-activated P-GSCs were washed with phosphate buffered saline (PBS, Corning) via centrifugation (6,000 rpm, 10 min) twice and were mixed with 0.2 mL of 1 mM cyclic RGD peptides (Selleck Chemicals LLC) or 1 mM cyclic RAD peptides (Anaspec) in PBS solution. The mixture was shaken for at least 16 hours. cRGD-conjugated P-GSCs (cRGD-GSCs) or cRAD-conjugated P-GSCs (cRAD-GSCs) were washed with PBS solution three times via centrifugation (6,000 rpm, 10 minutes).

Cell culture and in vitro cytotoxicity test of cRGD-GSCs. MCF 7 and MDA-MB 231 cancer cells were cultured in Dulecco`s Modified Eagle`s Medium (DMEM, Corning) containing 10 % fetal bovine serum (Corning) and 1% penicillin-streptomycin. The cancer cells were incubated at 37 °C in a 5% CO_2 incubator. For the cell viability test, MDA-MB 231 and MCF 7 cells were plated in a 96-well plate at a cell density of 8,000 cells per well. After 24 hours, cRGD-GSCs were added to each well by adjusting the construct concentration from 0 pM to 30 pM. The cells were incubated with cRGD-GSCs for 24 hours. The unbound cRGD-GSCs were removed by rinsing each well using the PBS solution. Each well was filled with 0.2 mL of DMEM media containing 3-(4,5-Dimethylthiazol-2yl)-2,5-Diphenyltetrazolium Bromide (0.5 mg mL^{-1}), followed by 3 hours-incubation. Each well was re-filled with 0.2 mL of dimethyl sulfoxide (DMSO, Sigma Aldrich) to dissolve the formazan salt. For the test of cell viability in each well, the absorbance at 570 nm was acquired using a well-plate reader (Synergy HT, BioTek).

***In vitro* PA cancer imaging using cRGD-GSCs, PEGylated P-GSCs, or cRAD-GSCs.** MCF 7 and MDA-MB 231 cancer cells were plated in a 6-well plate at a cell density of 100,000 cells per well. cRGD-GSCs (30 pM) in DMEM were added to each well, followed by incubation for

24 hours. Unbound cRGD-GSCs were rinsed out and labeled cancer cells were then collected via trypsinization. The cell concentration was adjusted to 1,200 cells μL^{-1} in PBS solution. The cell suspension in PBS was mixed with 16 % gelatin (Sigma Aldrich) aqueous solution. The gelatin mixture containing the labeled cancer cells was dropped onto a gelatin/silica phantom base, making a dome-shaped inclusion. The gelatin phantom was placed in the Vevo 2100/LAZR imaging system and then illuminated by laser pulses at 800 nm to image the cell-containing inclusions. PA gain and B-mode gain were set as 40 dB and 18 dB, respectively. US/PA images were acquired using a LZ250 ultrasound transducer.

For in vitro PA comparison of MDA-MB 231 cancer cells after labeling with cRGD-GSCs, PEGylated P-GSCs (PEG-GSCs), or cRAD-GSCs, MDA-MB 231 cancer cells were plated in a 6-well plate at a cell density of 100,000 cells per well. cRGD-GSCs, PEG-GSCs, cRAD-GSCs (30 pM) or cRGD-GSCs (30 pM) with free cRGD ligands (50 pM) as an inhibition control in DMEM were added to each well, followed by incubation for 24 hours (For the inhibition control experiment, free cRGD ligands were pre-incubated with cancer cells 30 minutes before cRGD-GSCs were injected). Unbound GSCs were rinsed out and labeled cancer cells were then collected via trypsinization. The cell concentration was adjusted to 1,200 cells μL^{-1} in PBS solution. The cell suspension in PBS was mixed with 16 % gelatin (Sigma Aldrich) aqueous solution. The gelatin mixture containing the labeled cancer cells was dropped onto a gelatin/silica phantom base, making a dome-shaped inclusion. The gelatin phantom was placed in the Vevo 2100/LAZR imaging system and then illuminated by laser pulses at 800 nm to image the cell-containing inclusions. PA gain and B-mode gain were set as 40 dB and 18 dB, respectively. US/PA images were acquired using a LZ250 ultrasound transducer.

***In vivo* PA imaging using cRGD-GSCs.** *In vivo* experiments were carried out under the Institutional Animal Care and Use Committee (IACUC) guidelines of the Georgia Institute of

Technology (Approval number is A100281). For *in vivo* experiments, we utilized an LZ550 ultrasound transducer. *In vivo* PA signal amplitude and contrast from cRGD-GSCs at 700-900 nm wavelengths were measured using the Vevo2100/LAZR imaging system after subcutaneous injection of the cRGD-GSCs (170 pM) to the flank of mice. PA gain and B-mode gain were set as 40 dB and 18 dB, respectively.

For the *in vivo* PA cancer imaging application, MDA-MB 231 cells (50 μL, 1,000,000 cells) were subcutaneously injected into the flank of mice (The Jackson Laboratory, 6-8 weeks old, female, NU/J). Once tumor volumes reached at least 100 mm^3, measured by calipers, 50 μL of cRGD-GSCs (850 pM) was systemically administrated to the mice via tail-vein injection. As a control group, saline solutions were injected via the tail-vein in a second group of mice. After 24 hours, US/PA images of the tumor were acquired using the Vevo2100/LAZR system within the 700-900 nm spectral range with 2 nm intervals. PA gain and B-mode gain were set as 40 dB and 18 dB, respectively.

Supplementary Note 3. Supplementary Figures

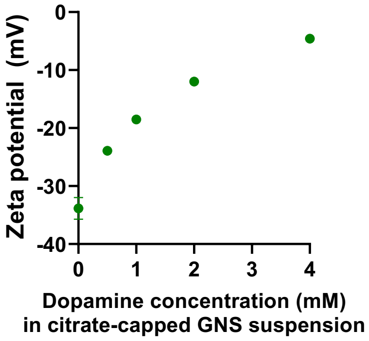

Figure S1 | Alterations in surface charges of citrate-functionalized GNSs in the presence of dopamine molecules with different concentrations (n=3). Data are presented as the mean ± standard deviation.

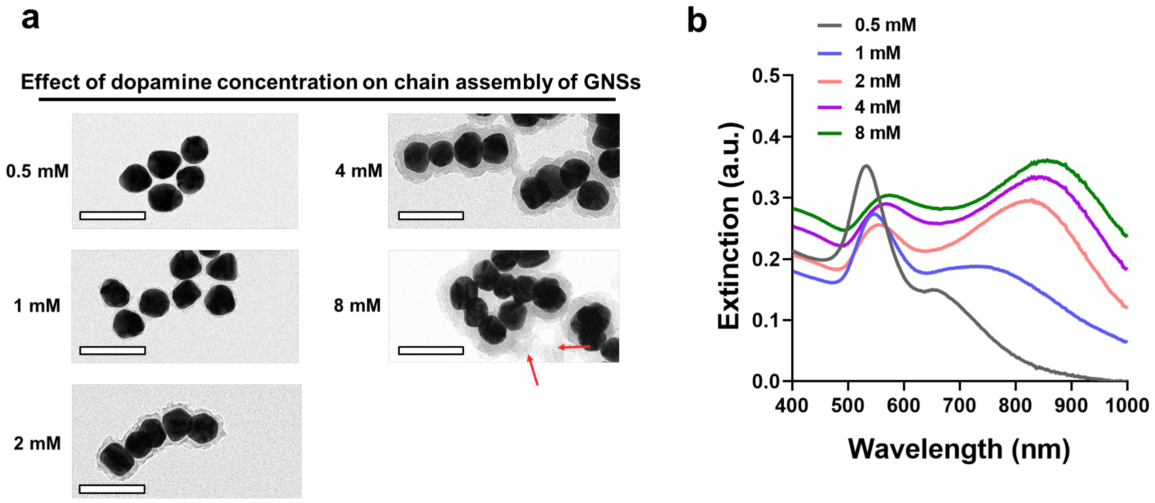

Figure S2 | Synthesis of P-GSCs at different dopamine concentrations. a) TEM image of P-GSCs fabricated at the different dopamine concentrations in the chain assembly process. The scale bars are 100 nm. Red arrows indicate self-nucleated polydopamine particles on P-GSCs. b) Corresponding UV-vis-NIR spectrum of the P-GSCs.

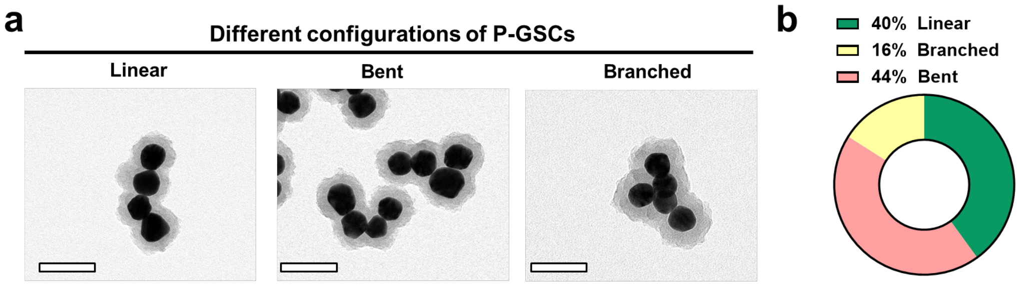

Figure S3 | a) TEM images for P-GSCs with different configurations, including linear, bent, and branched morphologies. Scale bars are 100 nm. b) Analysis of the configuration populations (n=50).

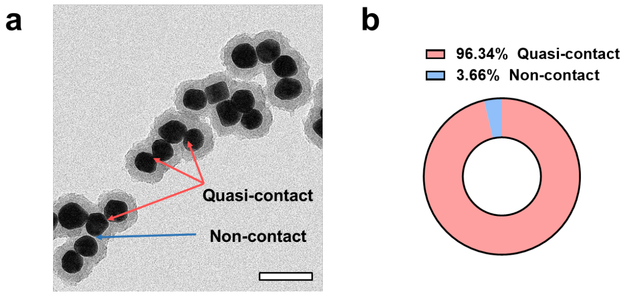

Figure S4 | a) TEM images of P-GSCs showing the contact between neighboring GNSs. Scale bars are 100 nm. b) Analysis of the GNS contact populations (n=82).

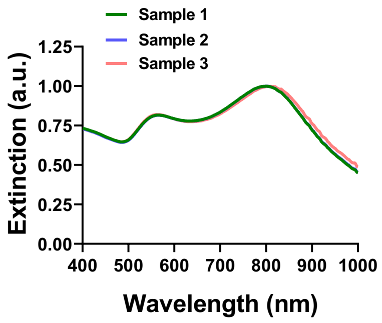

Figure S5 | UV-vis-NIR spectra of P-GSCs fabricated from different batches. The consistency of optical spectra of the P-GSCs ensures the synthesis reproducibility.

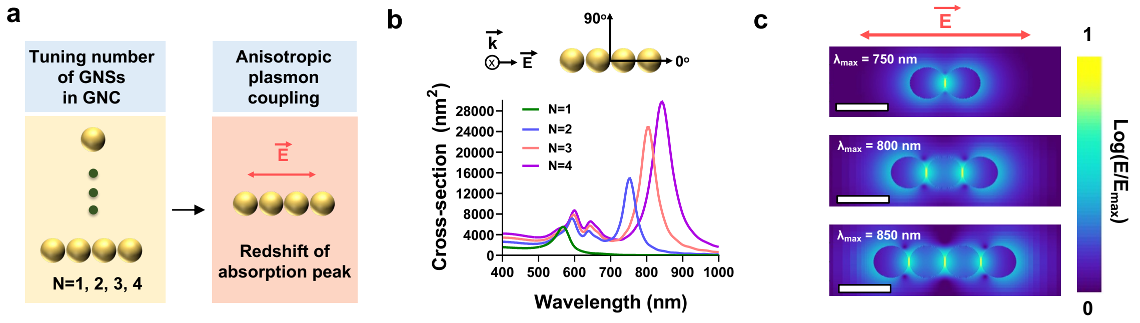

Figure S6 | FDTD simulation insights on P-GSC optical absorption at NIR wavelengths. a) Optical absorption response mechanism of P-GSCs, using a simplified model to simulate the effect of varying the GNS count (1 to 4) in a chain on NIR absorption intensity. The simulation assumes a polydopamine refractive index, reflecting its full coverage of GSC surfaces. b) Absorption cross-section enhancement and redshift towards NIR with increasing GNS numbers per GSC, demonstrating enhanced NIR absorption capability. c) Near electric-field distribution for GSCs with 2 to 4 GNSs, highlighting the local field enhancement at GNS gap junctions, highlighting the near-field coupling and plasmon mode hybridization's role in the strong NIR optical absorption of GSCs.

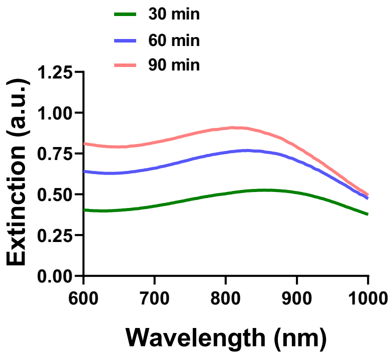

Figure S7 | UV-vis-NIR spectra of P-GSCs versus the reaction time for dopamine polymerization. For this experiment, the chain concentration was adjusted to 30 pM.

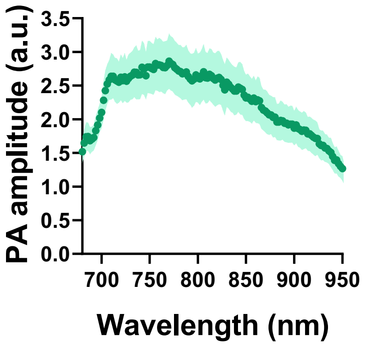

Figure S8 | PA signal generation from P-GSCs with a polymer layer within the 680-950 nm spectral range (n=5). The chain concentration was adjusted to 100 pM. Data are presented as the mean ± standard deviation.

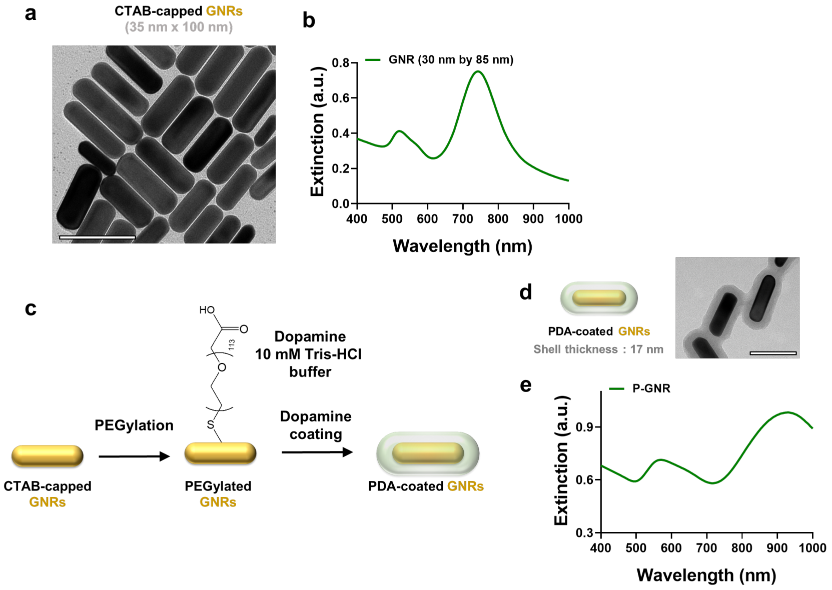

Figure S9 | Synthesis of P-GNRs. a) TEM image of GNRs (35 nm by 100 nm). b) Corresponding UV-vis-NIR spectrum of the GNRs. c) Strategy to fabricate P-GNRs. d) TEM image of P-GNRs. e) Corresponding UV-vis-NIR spectrum of the P-GNRs. The scale bars are 100 nm.

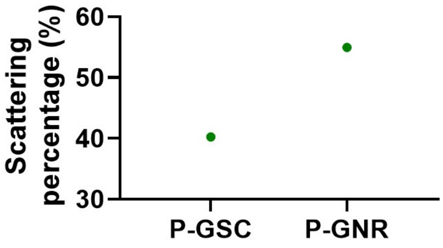

Figure S10 | Calculated scattering percentages of P-GSC and P-GNR at their peak absorption wavelength via the FDTD simulation.

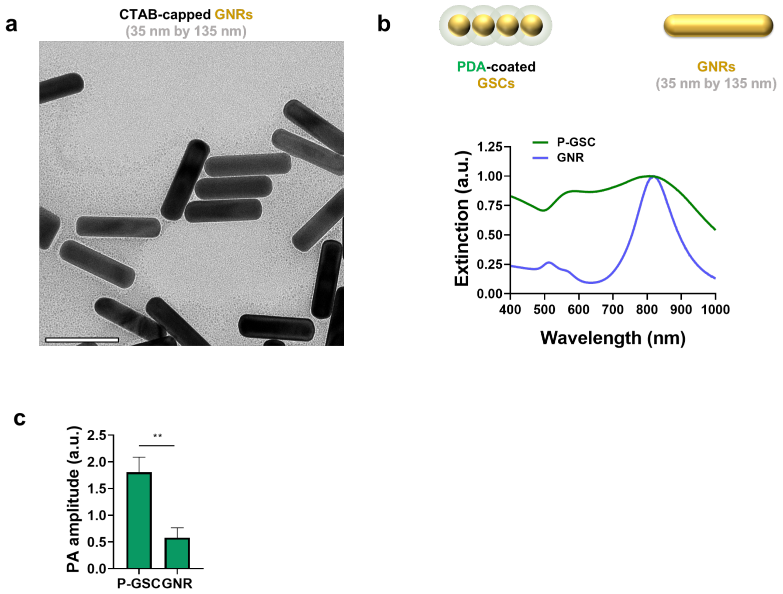

Figure S11 | Synthesis of GNRs. a) TEM image of GNR (35 nm by 135 nm). The scale bar is 100 nm. b) Corresponding UV-vis-NIR spectrum of the GNRs, comparing it with that of P-GSCs. c) A comparison of PA signal generation between P-GSCs and GNRs (n=3). Data are presented as the mean ± standard deviation. The statistical analysis for Figure S7c was conducted using a two-tailed Student's t-test. The statistically significant difference is represented as the asterisk (**: $p < 0.01$).

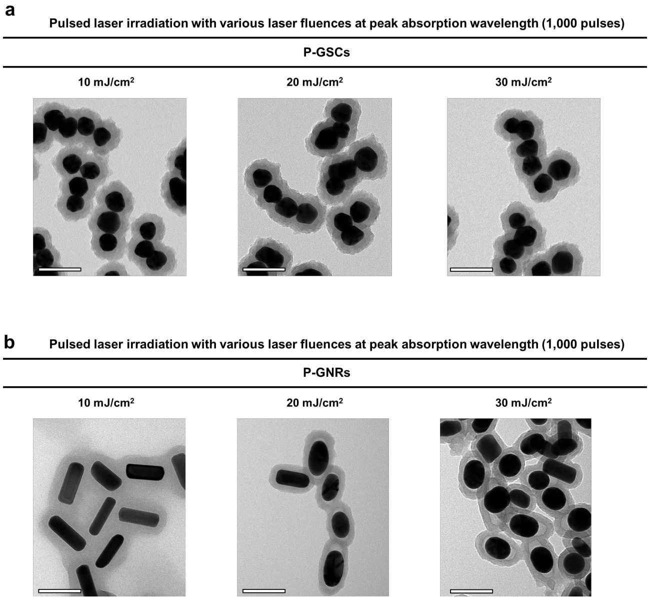

Figure S12 | A comparison of the photodamage threshold between P-GSCs and P-GNRs. a, b) TEM images of P-GSCs and P-GNRs after pulsed laser illumination with different laser fluences, including 10 mJ cm^{-2}, 20 mJ cm^{-2}, and 30 mJ cm^{-2}.

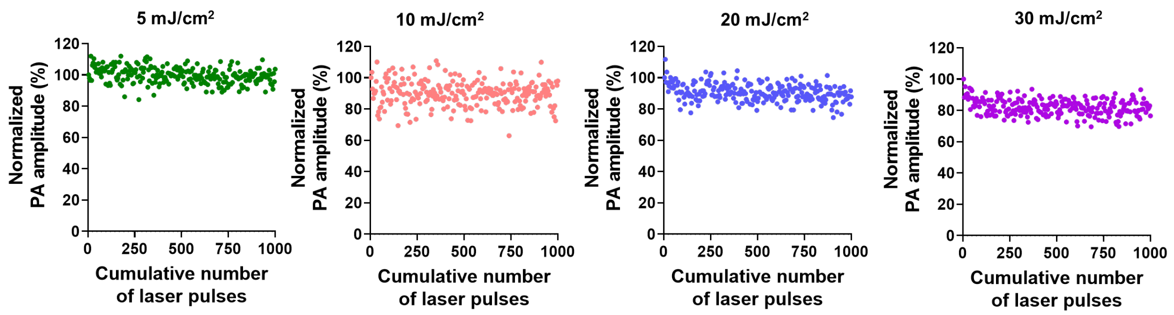

Figure S13 | PA signal generation from P-GSCs at different laser fluences.

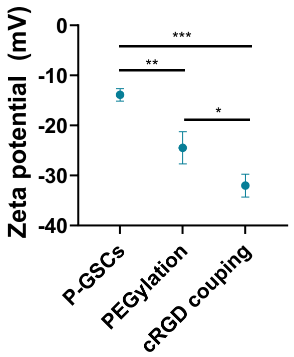

Figure S14 | Zeta potential analysis to investigate the serial surface functionalization of P-GSCs in the cRGD coupling process (n=3). Data are presented as the mean ± standard deviation. The statistical analysis was conducted through one-way analysis of variance (ANOVA) using Tukey post-hoc test. The statistically significant differences are represented as the asterisks (*: $p < 0.05$; **: $p < 0.01$; ***: $p < 0.001$).

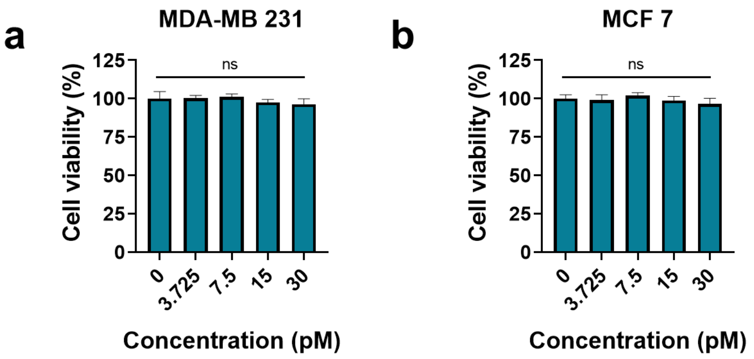

Figure S15 | Cell viability of MDA-MB 231 and MCF 7 cells after 24 hours-incubation with cRGD-GSCs at different construct concentrations (n=3). Data are presented as the mean ± standard deviation. The statistical analysis was conducted through one-way analysis of variance (ANOVA) using Tukey post-hoc test. The statistically non-significant differences are represented as ns.

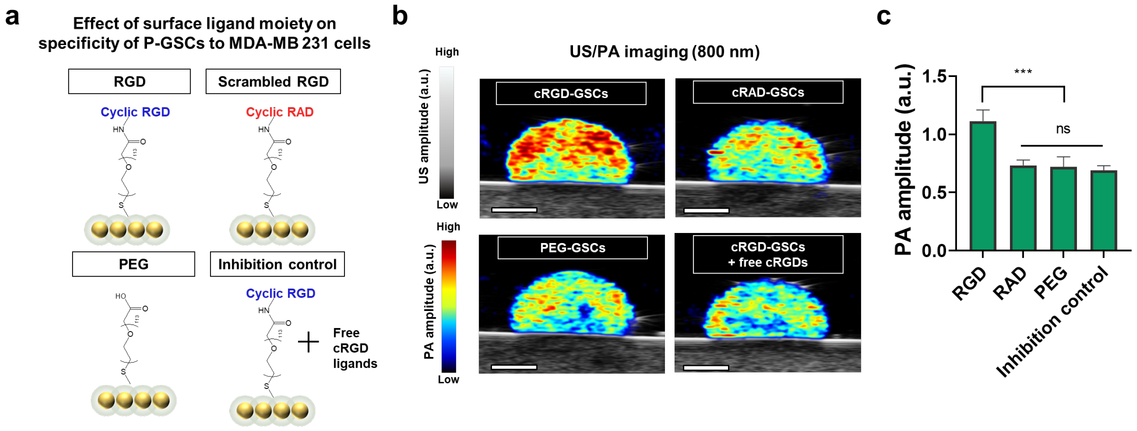

Figure S16 | a) Schematic illustrations of P-GSCs with different surface ligand moieties, including cyclic RGDs, scrambled cyclic RGDs (cyclic RADs), and PEG molecules. As an inhibition control to investigate the effect of surface ligand moiety on target specificity to MDA-MB 231 cancer cells, cRGD-GSCs and free cRGD ligands were co-incubated with MDA-MB 231 cells. b) US/PA images (800 nm wavelength) of dome phantoms containing labeled MDA-MB 231 cancer cells at the cell density of 600 cells μL^{-1} and c) corresponding quantification of PA amplitude for each dome phantom (n=3). The scale bars are 2 mm. The imaging experiments were repeated independently three times and similar results were obtained. Data are presented as the mean ± standard deviation. The statistical analysis was conducted through one-way analysis of variance (ANOVA) using Tukey post-hoc test. The statistically significant differences are represented as the asterisks ***: $p < 0.001$). The statistically non-significant difference is represented as ns.

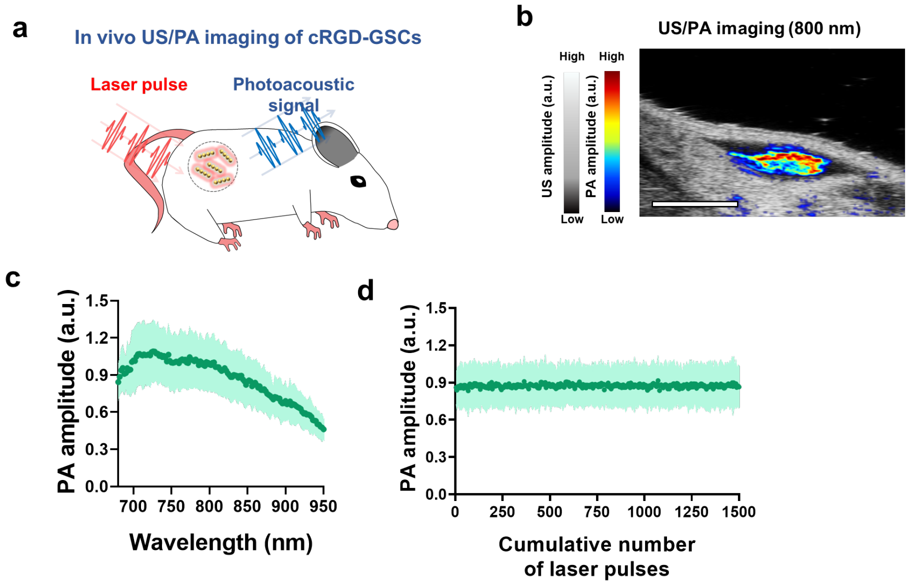

Figure S17 | *In vivo* PA signal generation from P-GSCs. a) Schematic illustration of *in vivo* PA imaging of P-GSCs after subcutaneous injection. b) US/PA image of P-GSCs (170 pM) subcutaneously injected to the flank of mouse. c) Corresponding PA spectra of P-GSCs (n=3). d) PA signal from P-GSCs for 1,500 laser pulses at a laser fluence of 10 mJ cm^{-2} (n=3). Data are presented as the mean ± standard deviation. The imaging experiments were repeated independently three times and similar imaging results were received.

Table S1 | Photodamage thresholds of the existing plasmonic nanoparticles under pulsed laser illumination

Nanoparticle (dimension)	Laser wavelength	Photodamage threshold	Ref.
Gold nanorod (25 nm by 85 nm)	*803 nm*	*11 mJ cm*$^{-2}$	[17]
Gold nanoplate (100 nm)	*1064 nm*	*10 mJ cm*$^{-2}$	[18]
Gold nanostars (100 nm)	*1064 nm*	*15 mJ cm*$^{-2}$	[19]
Gold-silica core-shell nanorod (12 nm by 28 nm)	*800 nm*	*12 mJ cm*$^{-2}$	[20]

References

(1) Chen, Y.-S.; Frey, W.; Aglyamov, S.; Emelianov, S. Environment-Dependent Generation of Photoacoustic Waves from Plasmonic Nanoparticles. *Small* **2012**, *8* (1), 47–52.

(2) Chen, Y.-S.; Zhao, Y.; Yoon, S. J.; Gambhir, S. S.; Emelianov, S. Miniature Gold Nanorods for Photoacoustic Molecular Imaging in the Second Near-Infrared Optical Window. *Nat. Nanotechnol.* **2019**, *14* (5), 465–472. https://doi.org/10.1038/s41565-019-0392-3.

(3) Shahbazi, K.; Frey, W.; Chen, Y.-S.; Aglyamov, S.; Emelianov, S. Photoacoustics of Core–Shell Nanospheres Using Comprehensive Modeling and Analytical Solution Approach. *Commun Phys* **2019**, *2* (1), 119. https://doi.org/10.1038/s42005-019-0216-7.

(4) Mantri, Y.; Jokerst, J. V. Engineering Plasmonic Nanoparticles for Enhanced Photoacoustic Imaging. *ACS Nano* **2020**, *14* (8), 9408–9422. https://doi.org/10.1021/acsnano.0c05215.

(5) Johnson, P. B.; Christy, R. W. Optical Constants of the Noble Metals. *Phys. Rev. B* **1972**, *6* (12), 4370–4379. https://doi.org/10.1103/PhysRevB.6.4370.

(6) Kim, M.; Lee, J.; Nam, J. Plasmonic Photothermal Nanoparticles for Biomedical Applications. *Adv. Sci.* **2019**, *6* (17), 1900471. https://doi.org/10.1002/advs.201900471.

(7) Rajabpour, A.; Seif, R.; Arabha, S.; Heyhat, M. M.; Merabia, S.; Hassanali, A. Thermal Transport at a Nanoparticle-Water Interface: A Molecular Dynamics and Continuum Modeling Study. *The Journal of Chemical Physics* **2019**, *150* (11), 114701. https://doi.org/10.1063/1.5084234.

(8) Baffou, G.; Quidant, R. Thermo-Plasmonics: Using Metallic Nanostructures as Nano-Sources of Heat. *Laser Photonics Rev.* **2013**, *7* (2), 171–187.

(9) Baffou, G.; Rigneault, H. Femtosecond-Pulsed Optical Heating of Gold Nanoparticles. *Phys. Rev. B* **2011**, *84* (3), 035415. https://doi.org/10.1103/PhysRevB.84.035415.

(10) Kim, M.; VanderLaan, D.; Lee, J.; Choe, A.; Kubelick, K. P.; Kim, J.; Emelianov, S. Y. Hyper-Branched Gold Nanoconstructs for Photoacoustic Imaging in the Near-Infrared Optical Window. *Nano Lett.* **2023**, *23* (20), 9257–9265. https://doi.org/10.1021/acs.nanolett.3c02177.

(11) Wang, H.; Chen, Y.-S.; Zhao, Y. Understanding the Near-Field Photoacoustic Spatiotemporal Profile from Nanostructures. *Photoacoustics* **2022**, *28*, 100425. https://doi.org/10.1016/j.pacs.2022.100425.

(12) Turkevich, J.; Stevenson, P. C.; Hillier, J. A Study of the Nucleation and Growth Processes in the Synthesis of Colloidal Gold. *Discuss. Faraday Soc.* **1951**, *11*, 55. https://doi.org/10.1039/df9511100055.

(13) Kimling, J.; Maier, M.; Okenve, B.; Kotaidis, V.; Ballot, H.; Plech, A. Turkevich Method for Gold Nanoparticle Synthesis Revisited. *J. Phys. Chem. B* **2006**, *110* (32), 15700–15707. https://doi.org/10.1021/jp061667w.

(14) Bastús, N. G.; Comenge, J.; Puntes, V. Kinetically Controlled Seeded Growth Synthesis of Citrate-Stabilized Gold Nanoparticles of up to 200 Nm: Size Focusing versus

Ostwald Ripening. *Langmuir* **2011**, *27* (17), 11098–11105. https://doi.org/10.1021/la201938u.

(15) Ye, X.; Zheng, C.; Chen, J.; Gao, Y.; Murray, C. B. Using Binary Surfactant Mixtures To Simultaneously Improve the Dimensional Tunability and Monodispersity in the Seeded Growth of Gold Nanorods. *Nano Lett.* **2013**, *13* (2), 765–771. https://doi.org/10.1021/nl304478h.

(16) Repenko, T.; Rix, A.; Nedilko, A.; Rose, J.; Hermann, A.; Vinokur, R.; Moli, S.; Cao-Milàn, R.; Mayer, M.; Von Plessen, G.; Fery, A.; De Laporte, L.; Lederle, W.; Chigrin, D. N.; Kuehne, A. J. C. Strong Photoacoustic Signal Enhancement by Coating Gold Nanoparticles with Melanin for Biomedical Imaging. *Adv. Funct. Mater.* **2018**, *28* (7), 1705607. https://doi.org/10.1002/adfm.201705607.

(17) Knights, O. B.; Ye, S.; Ingram, N.; Freear, S.; McLaughlan, J. R. Optimising Gold Nanorods for Photoacoustic Imaging *in Vitro*. *Nanoscale Adv.* **2019**, *1* (4), 1472–1481. https://doi.org/10.1039/C8NA00389K.

(18) Luke, G. P.; Bashyam, A.; Homan, K. A.; Makhija, S.; Chen, Y.-S.; Emelianov, S. Y. Silica-Coated Gold Nanoplates as Stable Photoacoustic Contrast Agents for Sentinel Lymph Node Imaging. *Nanotechnology* **2013**, *24* (45), 455101. https://doi.org/10.1088/0957-4484/24/45/455101.

(19) Khanadeev, V. A.; Kushneruk, S. A.; Simonenko, A. V.; Akchurin, G. G.; Akchurin, G. G.; Tuchin, V. V.; Khlebtsov, N. G. Nanosecond Laser-Induced Photomodification of Gold Nanostars of Various Sizes. In *Saratov Fall Meeting 2019: Laser Physics, Photonic Technologies, and Molecular Modeling*; Derbov, V. L., Ed.; SPIE: Saratov, Russian Federation, 2020; p 21. https://doi.org/10.1117/12.2563977.

(20) Chen, Y.-S.; Frey, W.; Kim, S.; Homan, K.; Kruizinga, P.; Sokolov, K.; Emelianov, S. Enhanced Thermal Stability of Silica-Coated Gold Nanorods for Photoacoustic Imaging and Image-Guided Therapy. *Opt. Express* **2010**, *18* (9), 8867. https://doi.org/10.1364/OE.18.008867.
